# The Phenomenon of T3SS Inactivation for *Pseudomonas aeruginosa* Strains from a Chronic Infection Locus: Do Mutations in T3SS-Regulators Matter?

**DOI:** 10.1128/spectrum.00494-22

**Published:** 2022-05-16

**Authors:** T. Savinova, Y. Bocharova, N. Mayanskiy, I. Chebotar

**Affiliations:** a Pirogov Russian National Research Medical University, Moscow, Russia; University of Pittsburgh School of Medicine

**Keywords:** *Pseudomonas aeruginosa*, cystic fibrosis, type III secretion

## LETTER

In some chronic infections, Pseudomonas aeruginosa is a key pathogen determining the course of the disease. The type III secretion system (T3SS) is an important virulence factor of P. aeruginosa which can be inactivated under chronic conditions, such as cystic fibrosis (CF) (1). In a recent study by Karash et al. (2), the authors described the impact of several regulatory proteins on T3SS gene expression in two P. aeruginosa strains isolated from a chronic cutaneous ankle wound in a patient with keratitis-ichthyosis-deafness (KID) syndrome. They hypothesized that T3SS inactivation might promote the persistence of P. aeruginosa in the chronic infection locus. In these two KID isolates, the *exsA*, *vfr*, and *cyaB* gene sequences were identical to those in a reference strain, whereas *fimV* and *bifA* contained frameshift mutations (2).

This article attracted our attention while we were examining the T3SS genes in 88 P. aeruginosa genomes recovered from CF patients from 29 regions in Russia using whole-genome sequencing (GenBank, BioProject accession numbers PRJNA786945, PRJNA770198). In these isolates, we analyzed genes involved in the T3SS contact toxin and protein synthesis and regulation in the context of Karash’s hypothesis of their regulatory role in T3SS inactivation. The target gene sequences were compared by BLASTn using megablast default parameters (expect threshold = 0.05, word size = 28, match/mismatch scores = 1, −2, gap cost = linear, filter = low complexity regions). The T3SS gene sequences from the ATCC 27853 and PAO1 strains were used as the reference.

Analysis of the T3SS genes is presented in Table 1. All examined P. aeruginosa isolates contained frame-disrupting (in-frame and out-of-frame indels) mutations in one or more T3SS structural genes. None of the 88 isolates possessed a set of T3SS structural genes which was identical to the reference or carried only synonymous or missense mutations.

**TABLE 1:** Mutations in the T3SS-related genes among 88 cystic fibrosis P. aeruginosa genomes

Gene	Absent in *N* isolates	Isolates with missense mutations^*a*^ (*n*)	Isolates with frameshift mutations (*n*)	
			Out-of-frame indels^*b*^	In-frame indels^*c*^
Effector protein genes of the T3SS complex				
* exoU* ^*d*^	70	14	0	0
* exoS*	3^*e*^	18	1	17
* exoT*	3^*e*^	79	3	0
* exoY*	6^*e*^	67	12	1
				
Structural genes of the T3SS complex^*f*^	3^*e*^	85	6	83
				
Regulatory genes for T3SS				
* exsA*	3^*e*^	12	0	0
* cyaB*	0	22	0	0
* vfr*	0	2	2	0
* fimV*	0	49	1	38
* bifA*	0	9	10	1

a Mutations leading to 1 to 4 amino acid substitutions with unclear significance.

b Mutation resulting in stop codons, frameshifts, or premature termination of translation.

c Mutations leading to insertion or deletion of ≥3 amino acids.

d *exoU* from NCTC 11447 was used as the reference sequence.

e The complete set of T3SS effector protein genes, structural genes, and *exsA* was missed in 3/88 isolates.

f In total, 32 structural genes of T3SS were analyzed.

Next, we analyzed the gene sequences that Karash et al. (2) considered important for T3SS regulation, including *exsA* (primary activation of T3SS transcription), *vfr* (critical activation of *exsA* expression), *cyaB* (activation of *exsA* expression), *fimV* (regulation of adenylate cyclase CyaB), and *bifA* (regulation of cyclic-di-GMP phosphodiesterase). In *exsA* and *cyaB*, only synonymic substitutions and one or two amino acid substitution-inducing mutations were found. In 1 of the 88 isolates, the *vfr* gene harbored a frameshift mutation leading to a premature stop codon; another isolate contained a 5-bp deletion which resulted in a frameshift in *vfr*. The remaining 83/88 *vfr* sequences were either wild-type or contained synonymous substitutions or one amino acid substitution-inducing mutation. The *bifA* gene was more altered, harboring 11 significant mutations, including three nonsense mutations, seven out-of-frame deletions and insertions, and one in-frame deletion. In 1/88 isolates, a nonsense mutation was discovered in *fimV*. In addition, in 35/88 genomes, *fimV* possessed 9- or 15-nucleotide insertions with putative roles in regulator inactivation.

Among our collection of 88 CF isolates, 56 different sequence types (STs), including 7 novel STs, were detected. The five most prevalent STs included ST235 (*n* = 8), ST274 (*n* = 4) and its double-locus variant ST2554 (*n* = 5), ST17 (*n* = 3), and ST395 (*n* = 3). Other STs were represented by one or two isolates. Thus, Pseudomonas aeruginosa exhibits a typical epidemic population structure characterized by a diversity of unrelated sequence types with the predominance of a small number of high-epidemic risk clones.

Overall, 40 of 88 isolates harbored a set of T3SS regulatory genes that were identical to the reference or carried only synonymous or missense mutations.

We agree with the conclusions of Karash et al. (2) regarding mutation-dependent inactivation of T3SS-system regulators, which is essential for understanding contact toxin machinery. Our study complements the data on the prevalence of various mutations in T3SS genes and demonstrates the real scale of the mutational downregulation for T3SS-regulating proteins among CF P. aeruginosa clinical isolates.

### Data availability.

The whole-genome sequences examined in our study are available from BioProject under the accession numbers PRJNA786945 and PRJNA770198.
